# Methicillin-resistant *Staphylococcus aureus* (MRSA) nasal carriage among patients with diabetes at the Korle Bu Teaching Hospital

**DOI:** 10.1371/journal.pone.0257004

**Published:** 2021-09-17

**Authors:** Ramzy B. Anafo, Yacoba Atiase, Fleischer C. N. Kotey, Nicholas T. K. D. Dayie, Patience B. Tetteh-Quarcoo, Samuel Duodu, Mary-Magdalene Osei, Khalid J. Alzahrani, Eric S. Donkor

**Affiliations:** 1 Department of Medical Microbiology, University of Ghana Medical School, Korle Bu, Accra, Ghana; 2 Department of Medicine, University of Ghana Medical School, Korle Bu, Accra, Ghana; 3 FleRhoLife Research Consult, Teshie, Accra, Ghana; 4 Department of Biochemistry, Cell and Molecular Biology and West African Centre for Cell Biology of Infectious Pathogens, University of Ghana, Legon, Accra, Ghana; 5 Department of Clinical Laboratory Sciences, College of Applied Medical Sciences, Taif University, Taif, Saudi Arabia; Indiana University School of Medicine-Northwest, UNITED STATES

## Abstract

**Aim:**

To investigate the epidemiology of *S*. *aureus* and MRSA nasal carriage among people with diabetes at the Korle Bu Teaching Hospital in Accra, including the prevalence, predictors of carriage, and antibiotic resistance.

**Methodology:**

This study was cross-sectional, involving 300 diabetes patients and 106 non-diabetic individuals. Swab specimens of the nares were obtained from the participants and bacteriologically-cultured. Identification and characterization of *S*. *aureus* and MRSA were based on standard bacteriological methods; antimicrobial susceptibility testing was by the Kirby-Bauer method.

**Results:**

The prevalence of staphylococcal carriage, the diabetes group relative to the non-diabetes group, were 31.0% and 10.4% (*S*. *aureus*), and 3.3% and 0.0% (MRSA). Presence of diabetes predisposed to *S*. *aureus* carriage, but not MRSA nor coagulase-negative staphylococci (CoNS) carriage (*OR* = 3.88; *p* < 0.0001). Colonization with CoNS was protective of *S*. *aureus* (*OR* = 0.039, *p <* 0.001) and MRSA (*OR* = 0.115, *p =* 0.043) colonization among the diabetics. The antimicrobial resistance patterns recorded among the *S*. *aureus* isolated from the diabetic individuals relative to the non-diabetics were as follows: penicillin (95% vs. 91%), tetracycline (37% vs. 27%), cotrimoxazole (30% vs. 36%), erythromycin (17% vs. 0%), norfloxacin (13% vs. 0%), clindamycin (12% vs. 0%), gentamicin (9% vs. 0%), fusidic acid (10% vs. 9%), linezolid (4% vs. 0%), and rifampicin (5% vs. 0%). The proportion of multidrug resistant *S*. *aureus* was 41% (*n* = 38) in the diabetes group and 0% in the non-diabetes group; this difference was statistically significant (*p* = 0.01).

**Conclusions:**

The presence of diabetes predisposed the participants to *S*. *aureus* carriage by almost four folds, but not MRSA carriage. Colonization with CoNS was protective of *S*. *aureus* and MRSA carriage in the diabetes group. Finally, linezolid remains a good therapeutic agent for anti-MRSA therapy.

## Introduction

*Staphylococcus aureus* (*S*. *aureus*) is concurrently a commensal and a human pathogen [1,2]. Its pathogenic nature is seen in its implication in infections such as meningitis, septicaemia, pneumonia, endocarditis and osteomyelitis [2]. A key predisposing factor to these infections is carriage of the pathogen on the human body, which could occur on the skin, perineum, pharynx, gastrointestinal tract, vagina, or the axillae, but more frequently in the anterior nares, and thus making the anterior nares the predominant antecedent to invasive *S*. *aureus* infections [2–4]. Consequently, two broad categories of *S*. *aureus* carrier states have been identified–persistent carriage, which occurs in about a fifth of the general population, and intermittent carriage which occurs in about a third [5–7]. Non-carriers, who comprise about half of the general population, have been presumed to be resistant to *S*. *aureus* carriage [5–8].

Based on their susceptibility to methicillin, *S*. *aureus* strains have been identified as methicillin-resistant *S*. *aureus* (MRSA) and methicillin-susceptible *S*. *aureus* (MSSA). The methicillin resistance trait simultaneously confers on the organisms resistance to all beta-lactam antibiotics [9–11]. Like most other pathogens, *S*. *aureus* has additionally been classified into healthcare-associated, community-associated, and livestock-associated, based on the origin of infection; consequently, MRSA strains have also been categorized as healthcare-associated MRSA (HA-MRSA) [12], community-associated MRSA (CA-MRSA) [13–16], and livestock-associated MRSA (LA-MRSA) [17,18].

Factors that predispose to *S*. *aureus* carriage have been extensively studied, and have been noted to include non-ambulatory status, previous hospitalization, chronic haemodialysis, previous antibacterial therapy, previous MRSA infection or colonization, previous ICU admission, HIV infection, and diabetes mellitus [19–23]. Several studies have shown that many of the clinical infections with *S*. *aureus* arise by virtue of spread from healthy carriers, thus demonstrating that carrying out studies on predictors of *S*. *aureus* carriage is integral to understanding the potential for MRSA transmission and invasive infections [24,25]. MRSA poses an important public health threat as it is refractory to major antibiotic groups in routine use [26–28] and is a consistent etiology of outbreaks [29–31]. Besides, its infections are synchronous with extended hospital stays coupled with increased healthcare costs which could be as high as 44 million Euros [30–32].

Individuals with diabetes, 79% of whom are in low- and middle-income countries [33], have an increased risk of *S*. *aureus* and MRSA carriage [34–37]. Nasal carriage of *S*. *aureus* has been identified in several studies as an important pre-requisite for its infections [3,38]. Thus people with diabetes comprise an important risk group for *S*. *aureus* and MRSA carriage and infections. Yet, the determinants of *S*. *aureus* and MRSA carriage in this risk population have not been well studied. Most MRSA carriage studies appear to have focused on the general population [39,40] and a few risk groups, such as HIV-infected persons [41–43] and sickle cell disease patients [44]. In Africa, the prevalence of *S*. *aureus* and MRSA carriage among diabetic individuals, antibiogram of colonizing strains, as well as predictors of carriage of the pathogen are largely unknown. Such information would contribute to tailored prevention and control measures in this risk group and help reduce the morbidity and mortality associated with diabetes. Hence this study investigated the epidemiology of *S*. *aureus* and MRSA nasal carriage among diabetes patients at the Korle Bu Teaching Hospital (KBTH), including the relationship between diabetes and carriage of *S*. *aureus* and MRSA, predictors of carriage, and *S*. *aureus* antimicrobial resistance patterns.

## Materials and methods

### Study site, design, and sampling

This study was approved by the Ethical and Protocol Review Committee of the College of Health Sciences, University of Ghana (Unique identifier: “CHS-Et/M.3–9.16/2019-2020”), and the Institutional Review Board of KBTH (Unique identifier: “KBTH-STC/IRB/000144/2019”). Written informed consent was also obtained from all the participants. The study was carried out at the National Diabetes Management and Research Centre (NDMRC) and the Department of Surgery (specifically, the Ulcer Clinic), which are both located on the premises of the Korle Bu Teaching Hospital (KBTH). The NDMRC is a national resource centre for diabetes care, training, and research, offering mainly outpatient services. It is the largest diabetes centre in Ghana, and receives patients from hospitals and clinics around the country, as well as clinics and wards within the hospital. It has over 5000 registered patients and an average daily outpatient attendance of about 70–80 persons. Management of DFU at the NDMRC is often dependent on the patient and the ulcer; of interest is the location and stage of the ulcer, glycaemic control, the presence or otherwise of peripheral artery disease, and neuropathy. For plantar ulcers, offloading is encouraged; however the absence of offloading devices makes most patients non-adherent. With regard to the daily wound dressing, it is done with saline for clean wounds. As regards infected wounds, the cleaning is done with iodinated povidone and metronidazole solution; papain-urea ointment is applied when there is slough. Systemic antibiotics are also prescribed for infected wounds. There is addtionally serial surgical debridement on outpatient basis or admission for debridement in the theatre when needed–when peripheral artery disease is present, vascular surgeons are involved, but the cost of these procedures are often prohibitive for most patients. All these are done in addition to optimization of glucose control.

The study had a cross-sectional design, and involved sampling 300 diabetes patients and 106 non-diabetic individuals (serving as the control group) during the period January to June 2020. Recruitment of the diabetes patients was based on the following inclusion criteria: being a diabetes patient, and aged between 13 and 80 years. The non-diabetics were recruited based on their fasting/random blood sugar levels. Individuals with fasting blood sugar levels less than or equal to 7.0 mmol/l and less than or equal to 11.1 mmol/l for random blood sugar results (and not on glucose-lowering medications) were considered eligible for the study. The exclusion criteria for both the study and control groups were: refusal to participate, presence of severe disease (disease warranting hospitalization), and history of recent (two weeks) antimicrobial therapy.

After obtaining informed consent, information on possible predictors of colonization with *S*. *aureus* and MRSA (such as a history of pneumonia or tuberculosis [TB], owing to the association of the conditions with the respiratory tract) were gathered from the participants using a standard questionnaire, as well as anterior nasal swabs by a qualified physician, via rotation of sterile cotton swabs five times at the anatomical site. Each specimen was subsequently kept in an already-labeled 1 ml skim milk-tryptone-glucose-glycerin (STGG)-contained vial, and within four hours of collection, conveyed to the Department of Medical Microbiology, University of Ghana Medical School, for laboratory processing. The processing involved an intial two-minute vortexing and a subsequent storage at -80 ˚C, until needed.

### Laboratory analysis

Specimen processing, *S*. *aureus* and MRSA identification, antimicrobial susceptibility testing, and molecular investigations were done as previously described [43], with a few modifications. Media used in the culture of the specimens were blood, chocolate, MacConkey, and Mannitol salt agars. For each sample, all staphylococcal colonies with different morphologies were selected for follow-ups. Presumptive staphylococcal identification was with the aid of colonial morphology and reaction to Gram stain. Staphylococcal isolates that were coagulase-negative and -positive were respectively identified as coagulase-negative staphylococci (CoNS) and *S*. *aureus*. The latter were tested against penicillin (10 μg), tetracycline (30 μg), cefoxitin (30 μg), cotrimoxazole (1.25 μg trimethoprim + 23.75 μg sulphamethoxazole), erythromycin (15 μg), norfloxacin (10 μg), clindamycin (2 μg), gentamicin (10 μg), fusidic acid (10 μg), linezolid (10 μg), and rifampicin (5 μg), to determine their susceptibility, in accordance with Clinical and Laboratory Standards Institute (2020) guidelines. *S*. *aureus* ATCC 25923 was used as the control strain. Cefoxitin-resistant *S*. *aureus* were confirmed as *S*. *aureus* by means of polymerase chain reaction (PCR) amplification of the *nuc*A gene, and as MRSA through PCR amplicafication of the *mec*A gene.

During the molecular analyses, genomic DNA were extracted with the Zymo Research extraction kit (Zymo Research Corp., Irvine, USA) (as directed by the manufacturer) from an overnight lysogenic broth culture of each MRSA isolate, as well as that of an MRSA positive control strain. For each isolate, a mixture of extracted DNA (5 μL volume) and bromophenol blue gel loading buffer (2 μL volume) was separated using a 1.2% agarose gel electrophoresis, followed by UV-aided visualization of resultant bands (as a quality control measure). Subsequently, each extracted DNA served as a template for the *mecA* and *nucA* PCRs.

### Data analysis

The software STATA 14 (Strata Corp, College Station, TX, USA) was used for the data analysis. Besides using descriptive statistics to summarize demographic, clinical, and antimicrobial resistance data, univariate and multivariate analyses (including odds ratios and 95% confidence intervals, at a 0.05 alpha level) were used to identify predictors of colonization with *S*. *aureus* and MRSA.

### Ethical approval

This study was approved by the Ethical and Protocol Review Committee of the College of Health Sciences, University of Ghana (Unique identifier: “CHS-Et/M.3–9.16/2019-2020”), and the Institutional Review Board of KBTH (Unique identifier: “KBTH-STC/IRB/000144/2019”).

## Results

### Sociodemographic and clinical features of the participants

In total, four hundred and six (406) individuals–two hundred (200) diabetic individuals without foot ulcers, one hundred (100) diabetic individuals with foot ulcers, and one-hundred and six (106) non-diabetic individuals–participated in the study. Their sociodemographic data are presented in Table 1. Females were the majority in both groups, representing 74.7% (*n* = 224) and 50.9% (*n* = 54) among the diabetics and non-diabetics respectively. With regard to age, majority of the participants in the diabetes group were older than 60 years of age (53.7%, *n* = 161), whereas in the non-diabetes group, the 30–60 year old group had the most participants. The major type of residence inhabited by the participants of the diabetes group was self-contained apartments (54.0%, *n* = 162), whereas that of the non-diabetes group was compound houses (51.9%, *n* = 55). Furthermore, the participants in both study groups reported washing their hands with soap often [diabetes group (80.3%, *n* = 241); non-diabetes group (92.5%, *n* = 98)].

**Table 1:** Demographic and household characteristics of the study participants.

Demographic and household characteristics	Diabetics		Non-Diabetics	
	Number	%	Number	%
**Age (in years)**				
14–19	1	0.3	4	3.8
20–29	2	0.7	29	27.4
30–60	136	45.3	69	65.1
>60	161	53.7	4	3.8
**Gender**				
Male	76	25.3	52	49.1
Female	224	74.7	54	50.9
**Type of residence**				
Self-contained	162	54.0	51	48.1
Compound	138	46.0	55	51.9
**Number of individuals in household**				
<5	108	36.0	51	48.1
5–10 persons	179	59.7	51	48.1
11–20 persons	13	4.3	4	3.8
**Presence of health worker in household**				
Yes	74	24.7	53	50.0
No	225	75.0	53	50.0
**Hand washing with soap**				
Rarely	59	19.7	8	7.5
Often	241	80.3	98	92.5

Age (Diabetics) $\bar{(X},\;SD)$ = 60.73, 13.28 years; Age (Non-Diabetics) $\bar{(X},\;SD)$ = 37.26, 12.22 years; Number of individuals in household (Diabetics) $\bar{(X},\;SD)$ = 5.81, 2.75 persons; Number of individuals in household (Non-Diabetics) $\bar{(X},\;SD)$ = 5.14, 2.59 persons.

As regards the participants’ clinical features, none of the participants in the non-diabetes group had an history of hospitalization in the past year, history of pneumonia, or presence of foot ulcers, while 0.5% (*n* = 1) and 4.2% (*n* = 8) respectively had a history of tuberculosis and surgery. In the diabetes group, majority of the participants lacked a history of hospitalization in the past year (61.7%, *n* = 185), presence of foot ulcer (66.7%, *n* = 200), history of pneumonia (97.3%, *n* = 292), tuberculosis (98.7%, *n* = 296), and a history of surgery (58.7%, *n* = 176). Details of the clinical features are presented in Table 2.

**Table 2:** Clinical features of the study participants.

Clinical features	Diabetics		Non-Diabetics	
	Number	%	Number	%
* Self-reported self-medication				
Yes	109	36.3	39	20.6
No	191	63.7	67	35.4
**History of hospitalization in the past year**				
Yes	115	38.3	0	0.0
No	185	61.7	106	100.00
**Presence of foot ulcer**				
Yes	100	33.3	0	0
No	200	66.7	106	100
**History of pneumonia in the past year**				
Yes	8	2.7	0	0.0
No	292	97.3	106	100.00
**History of tuberculosis in the past year**				
Yes	4	1.3	1	0.5
No	296	98.7	105	99.5
**History of surgery in the past year**				
Yes	124	41.3	8	4.2
No	176	58.7	98	51.9

Number of hospitalizations (Diabetics) $\bar{(X},\;SD)$ = 0.61, 0.97; Number of hospitalizations (Non-Diabetics) $\bar{(X},\;SD)$ = 0.00, 0.00;

* Refers to participants’ reported self-medication with antibiotics and other medications within the past year.

### Relationship between diabetes and staphylococcal carriage

The prevalence of staphylococcal carriage, the diabetes group relative to the non-diabetes group, were 31.0% and 10.4% (*S*. *aureus*), and 3.3% and 0.0% (MRSA) (Table 3). Presence of diabetes was not significantly associated with MRSA carriage, but significantly associated with *S*. *aureus* carriage (OR = 3.88, *p* < 0.0001), with diabetes conferring an almost four-fold risk of *S*. *aureus* carriage. Eight of the *S*. *aureus* nasal carriers had concurrent *S*. *aureus* presence in their foot ulcers.

**Table 3:** A comparison of the study participants on their staphylococcal carriage.

Staphylococci	Diabetics	Non-Diabetics	OR (95% *CI*)	*p* value
	Prevalence (%)	Prevalence (%)		
*S*. *aureus*	31.0% (*n* = 93)	10.4% (*n* = 11)	3.88* (3. 89–7.59)	<0.0001
MRSA	3.3% (*n* = 10)	(*n* = 0)	–	–
CoNS	57.0% (*n* = 171)	46.2% (*n* = 49)	1.54 (0.99–2.41)	0.06

*Significant at 0.05 alpha level.

### Predictors of *S*. *aureus* and MRSA colonization among the diabetics and non-diabetics

Among the diabetics, colonization with coagulase-negative *Staphylococci* was protective of *S*. *aureus* (*OR* = 0.039, *p* < 0.001, 95% *CI* = 0.02–0.08) and MRSA colonization (*OR* = 0.115, *p* = 0.043, 95% *CI* = 0.014–932). However, no predictors of *S*. *aureus* and MRSA colonization were identified among the non-diabetics.

### Antimicrobial resistance patterns of the *S*. *aureus* isolates

**Fig 1:**
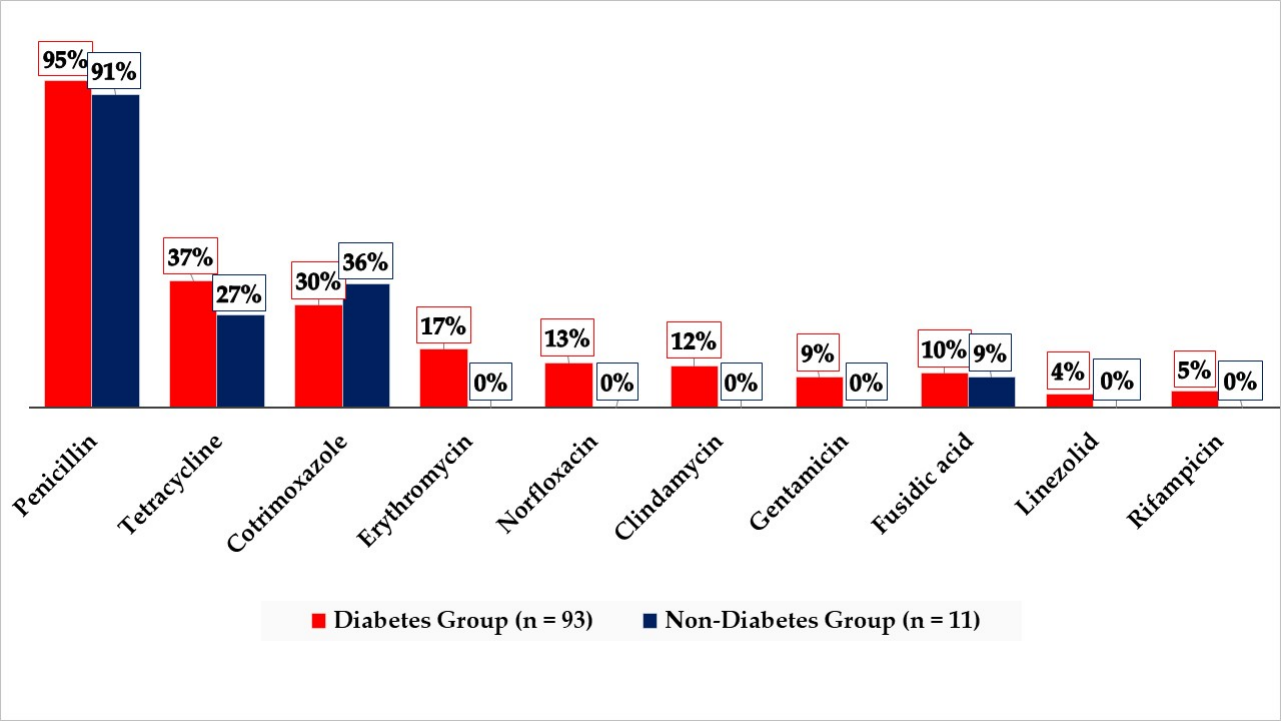


The highest proportion of *S*. *aureus* resistance to the various antimicrobials was recorded for penicillin (95% in the diabetes group and 91% in the non-diabetes group). Also, no resistance was recorded against any of linezolid, rifampicin, gentamicin, clindamycin, norfloxacin, and erythromycin in the non-diabetes group. In the diabetes group, the resistance rates recorded against each of these six antibiotics ranged between 4% and 17%. The resistance rates recorded against cotrimoxazole were 30% in the diabetes group and 36% in the non-diabetes group. None of the differences in these antibiotic resistance rates was statistically significant. However, as regards the proportion of multidrug resistance (MDR) (resistance to three or more antimicrobial classes, penicillin inclusive) among the *S*. *aureus* isolates, the diabetes group recorded 41% (*n* = 38), whereas the non-diabetes group recorded 0% (*n* = 0), and this difference was statistically significant (*p* = 0.01); even when penicillin was excluded from the determination of MDR, the MDR rate remained higher in the diabetes group (21%; *n* = 20) than in the control group (0%; *n* = 0), albeit not statistically significant (*p* = 0.09). Moreover, a comparison of the antibiotic resistance rates between the MRSA and MSSA isolates of the diabetes group demonstrated statistically significant differences for tetracycline (MRSA = 70% [7/10]; MSSA = 33% [*2*7/83]; *p* = 0.02), norfloxacin (MRSA = 4% [4/10]; MSSA = 10% [8/83]; *p* = 0.01), and gentamicin (MRSA = 30% (3/10); MSSA = 6% (5/83); *p* = 0.01), but not penicillin (MRSA = 0% [0/10]; MSSA = 94% [78/83]; *p* = 0.42), erythromycin (MRSA = 0% [0/10]; MSSA = 19% [16/83]; *p* = 0.13), clindamycin (MRSA = 0% [0/10]; MSSA = 13% [11/83]; *p* = 0.22), linezolid (MRSA = 0% [0/10]; MSSA = 5% [4/83]; *p* = 0.48), cotrimoxazole (MRSA = 40% [4/10]; MSSA = 29% [24/83]; *p* = 0.47), rifampicin (MRSA = 0% [0/10]; MSSA = 6.0% [5/83]; *p* = 0.42), nor fusidic acid (MRSA = 10% [1/10]; MSSA = 10% [8/83]; *p* = 0.97). A comparison of the rates of antimicrobial resistance of *S*. *aureus* between the diabetes and non-diabetes groups is presented in Fig 1.

## Discussion

This study appears to be the first to investigate the epidemiology of *S*. *aureus* and MRSA carriage among diabetics in Ghana, as well as one of the few MRSA carriage studies conducted among populations with diabetes in Africa and other parts of the world.

Individuals with diabetes had a higher odds for carriage of *S*. *aureus* (31.0% vs. 10.4%), but not MRSA (3.3% vs. 0.0%). It is important to note that this is not the first time that a risk population has been associated with a higher odds for *S*. *aureus* carriage, but not MRSA carriage. Donkor *et al*. [43] and Appiah *et al*. [44] made a similar observation among HIV-infected and sickle cell disease patients respectively in their studies conducted within the same geographical area as this study. This observation may be due to the low MRSA prevalence recorded by these studies (< 4%), as was also the occurrence in the current study, probably stemming from the generally-low MRSA prevalence in the country [45].

The *S*. *aureus* nasal carriage prevalence recorded in the current study falls within the nasal carriage prevalence of 8%–44.9% reported among other risk groups in the country [41,43,44]. It is also comparable to the 32.8% prevalence reported among a diabetic population in Egypt [46], and seems higher than that reported by Lin *et al*. [47] (8.7%) and Lin *et al*. [48] (16.4%) among two diabetic populations in China, but higher than those reported by Kutlu *et al*. [49], Ahluwalia *et al*. [50], and Saxena *al*. [51] respectively among diabetic populations in Turkey (41.8%), Australia (56.7%) and Saudi Arabia (72.4%). It needs to be pointed out, though, that the diabetic population sampled in the study of Ahluwalia *et al*. [50] were on hospital admission, and that of Saxena *et al*. [51] additionally had end-stage renal disease, and were receiving haemodialysis. These may account for their apparently higher *S*. *aureus* prevalence. The MRSA prevalence recorded in the current study however fell within the range of 0–8.5% reported in these studies [46–51].

Colonization with coagulase-negative *Staphylococci* was protective of *S*. *aureus* and MRSA colonization among the diabetics. This means absence of colonization with CoNS increased the odds of *S*. *aureus* and MRSA colonization. This is consistent with the reported inverse relationship between *S*. *aureus* and CoNS, which has been attributed to production of the *S*. *aureus*-cidal autoinducing peptide by CoNS [52–54].

The highest proportion of *S*. *aureus* resistance to the various antimicrobials was recorded for penicillin (95% in the diabetes group and 91% in the non-diabetes group). Owing to the wide usage of the antibiotic, previous studies in the country have also reported similar penicillin resistance rates among *S*. *aureus* [40,41,43,44]. These studies additionally recorded similar antimicrobial resistance rates for the other antimicrobials investigated [40,41,43,44]. Given the high resistance displayed against cotrimoxazole in the current study, it may be imperative to re-examine its usefulness as an antibiotic prophylaxis. Linezolid recorded low resistance rates, suggesting that it is still useful as an anti-MRSA agent. However, as it is one of the limited mainstays of anti-MRSA therapy, the 4% resistance rate recorded against the antimicrobial in the diabetes group underscores the need to step up campaigns against indiscriminate antimicrobial use. This recommendation is reinforced by the 40% MDR proportion recorded in the diabetes group.

Interpretation of the findings of this study is limited by absence of data on glycaemic control and grade and duration of DFU among the diabetics, as well as the relatively smaller number of non-diabetics. Besides, given the age-prevalent nature of diabetes, the majority of the participants in the diabetes group were older than 60 years of age, whereas in the non-diabetes group, the 30–60 year old group had the most participants. Also, the study lacks data on participants’ previous antimicrobial therapy in the context of agents, route of administration, and duration of therapy. Moreover, concordance of *S*. *aureus* carriage and foot ulcer infection could not be determined for the eight concomitant *S*. *aureus* carriers given the absence of genotypic data.

## Conclusion

The presence of diabetes predisposed the participants to *S*. *aureus* carriage by almost four folds, but not MRSA carriage. Moreover, colonization with CoNS was protective of *S*. *aureus* and MRSA carriage in the diabetes group. Finally, linezolid remains a good therapeutic agent for anti-MRSA therapy.

In light of the high proportion of multidrug resistant *S*. *aureus* in the diabetes group, it is necessary to continue carrying out MRSA surveillance studies among diabetic individuals and other risk groups for *S*. *aureus* and MRSA carriage.

## Supporting information

S1 File (DOCX)
