# Supplementary material for: Methicillin-resistant Staphylococcus aureus (MRSA) nasal carriage among patients with diabetes at the Korle Bu Teaching Hospital
Source: PLoS One. 2021 Sep 17;16(9):e0257004. doi: 10.1371/journal.pone.0257004 (PMC8448364; doi:10.1371/journal.pone.0257004)
Supplement: S1 File — (DOCX) [file pone.0257004.s001.docx]

##

## **MRSA PROJECT: QUESTIONNAIRE FOR ANALYSIS OF RISK FACTORS**

**SECTION A: BIODATA/DEMOGRAPHY**

1. Patient ID ………………
2. Age …….…………
3. Gender Male/Female

**SECTION B: HOUSEHOLD CHARACTERISTICS**

1. Type of Residence: Self-contained/Compound
2. How many people live in your household? ………………
3. How frequently do you wash your hands with soap? Rarely/Often

**SECTION C: MEDICAL HISTORY**

1. Have you self-medicated within the past year? Yes/No
2. Have you been hospitalized within the past year? Yes/No
3. If your answer to (8) is yes, how many times? …………
4. Do you have any co-morbidity? Yes/No
5. If your answer to (10) is yes, what condition is it? …..………
6. Do you have a foot ulcer? Yes/No
7. Have you had a history of pneumonia in the past year? Yes/No
8. Have you had a history of tuberculosis in the past year? Yes/No
9. Have you had a surgical procedure on your foot in the past year? Yes/No
10. Is any member of your household a health worker? Yes/No
